# Candidate Gene Identification and Genomic Prediction for Key Reproductive Traits in Yorkshire, Landrace, and Duroc Pigs

**DOI:** 10.3390/ani16070999

**Published:** 2026-03-24

**Authors:** Wenjie Hao, Wu-Sheng Sun, Zhuoshan Li, Jingbo Zhang, Lijun Shi, Hasi Chaolu, Qi Zhang, Teerath Kumar Suthar, Lixian Wang, Shu-Min Zhang

**Affiliations:** 1 College of Animal Science and Technology, Jilin Agricultural University, Changchun 130118, China; haowenjie@bestgenetics.com.cn (W.H.); sunwsh@jlau.edu.cn (W.-S.S.); sandy@bestgenetics.com.cn (Z.L.); jingbo881003@live.cn (J.Z.);; 2 Institute of Animal Science, Chinese Academy of Agricultural Sciences, Beijing 100193, China; 3 Chifeng Best Genetics Nucleus Biotech Group Co., Ltd., Chifeng 024000, China; hasichaolu@bestgenetics.com.cn

**Keywords:** pig, multi-breed, reproductive traits, GWAS, genomic prediction

## Abstract

The study analyzed ten reproductive traits in three major commercial breeds—Yorkshire, Landrace, and Duroc—raised under uniform management. Genetic parameters were estimated using a repeatability animal model in ASReml, genome-wide association studies (GWAS) were performed with 46,358 post-QC SNPs using GCTA, and genomic prediction was evaluated with GBLUP. Heritability estimates were low to moderate, with gestation length (GL) highest (0.33–0.41). GWAS identified significant loci across breeds: in Yorkshire, 37 genome-wide significant SNPs across 17 SSCs for seven traits; in Landrace, 16 SNPs for TNB and one for NBW; and in Duroc, 31 SNPs across 12 SSCs (predominantly for TNB). Among these SNPs, CNC10042060, CNC10160995, and CNCB10003799 were consistently associated with TNB in both Yorkshire and Duroc pigs. Additionally, five SNPs, CNC10012965, CNC10042060, CNC10120451, CNCB10003799, and CNCB10007759, showed significant associations with NBW in Yorkshire and Landrace pigs. Candidate genes mapped within ±1 Mb of significant SNPs were enriched for biologically plausible pathways. Genomic prediction accuracies ranged from low to high depending on trait and breed, such as reaching 0.68 for GL in 39 Yorkshire and 0.59 in Landrace. These results delineate shared and breed-specific genetic architectures, provide actionable markers and candidate genes, and can accelerate genetic gains in commercial breeding programs.

## Simple Summary

Reproductive performance is a key factor affecting productivity and profitability in pig breeding. Understanding the genetic basis of reproductive traits can help improve selection efficiency in commercial pig populations. In this study, ten reproductive traits were analyzed in three major commercial pig breeds—Yorkshire, Landrace, and Duroc—raised under the same management conditions. We estimated genetic parameters, identified genomic regions associated with these traits, and evaluated the potential of genomic prediction for breeding improvement. The results showed that most reproductive traits had low to moderate heritability, indicating that both genetic and environmental factors influence these traits. Genome-wide association analyses identified several significant genetic markers and candidate genes related to reproductive performance, including loci associated with total number born and newborn weight across breeds. In addition, genomic prediction showed promising accuracy for some traits, particularly gestation length. These findings improve our understanding of the genetic architecture of reproductive traits and provide useful genetic markers that may support genomic selection and accelerate genetic improvement in pig breeding programs.

## 1. Introduction

Reproductive performance is a major economic driver in pig production because it directly determines the number of marketable piglets and thereby influences farm productivity, herd turnover, and profitability [1,2]. Among commercial breeds, Yorkshire, Landrace, and Duroc are globally prominent [3] and exhibit distinct reproductive profiles: Yorkshire and Landrace excel in litter size traits, such as total number born (TNB) and number born alive (NBA), whereas Duroc contributes complementary superior offspring growth, making it integral to three-way crossbreeding schemes. Reported heritabilities for NBA differ modestly among breeds (Yorkshire 0.10; Duroc 0.09; Landrace 0.08) [4], underscoring heterogeneity in genetic architecture and the need for breed-tailored selection strategies.

Although heritability estimates for reproductive traits are abundant, most evidence is derived from Yorkshire populations [5,6,7,8], limiting generalizability across breeds. Genome-wide association studies (GWAS) have enabled the identification of quantitative trait loci (QTL) and candidate genes for complex reproductive traits [9,10,11,12,13]. However, most existing GWAS in Yorkshire populations have been conducted within isolated breed-specific contexts, where the detected QTLs are often confounded by breed-specific linkage disequilibrium (LD) patterns. Due to divergent selection histories, the LD phase between markers and causal mutations in Yorkshire may not be conserved in Landrace or Duroc. Consequently, markers significantly associated with reproductive traits in Yorkshire may fail to capture the same causal variants in other breeds, rendering single-breed-derived markers poorly transferable [14]. From a statistical perspective, single-breed GWAS are inherently underpowered to distinguish between truly pleiotropic variants and population-specific artifacts. Reproductive traits are low-heritability and polygenic, requiring large effective sample sizes to detect small-effect loci. Studies restricted to a single breed often suffer from limited sample diversity and fail to capture the allelic series that segregate differently across populations. This limitation leads to an incomplete catalog of causal variants, particularly those that may be rare or fixed in Yorkshire but segregated in Duroc or Landrace [15]. Methodological heterogeneity among previous GWAS—such as differences in SNP chip density, statistical models (e.g., single-marker regression vs. Bayesian approaches), and phenotype definitions—has resulted in inconsistent findings [16,17]. Therefore, by implementing a harmonized multi-breed GWAS framework, we aim to transcend these constraints, enabling the identification of both conserved QTLs that underpin core reproductive mechanisms and breed-specific variants that drive phenotypic divergence. This approach is essential for developing truly robust multi-breed genomic prediction models and for elucidating the genetic basis of breed-specific reproductive performance.

Traditional breeding strategies for pig reproductive traits depend primarily on long-term phenotypic recording and pedigree-based selection, which are constrained by several inherent limitations. Genomic prediction methods have been applied to reproductive traits to increase selection accuracy by capturing realized genomic relationships and marker effects [18,19,20], yet accuracies for reproductive traits still lag behind those for production traits owing to lower heritability, measurement noise, genotype-by-environment interactions, and limited transferability across breeds. Consequently, strategies that explicitly account for breed differences are needed to improve predictive performance across populations.

Hence, this study aims to estimate the identify genetic parameters and genomic prediction for reproductive traits in Yorkshire, Landrace, and Duroc pigs and to identify the candidate genes. This work will clarify the shared and breed-specific genetic architecture of reproduction, identify actionable genomic markers, and provide prediction results to accelerate genetic gain, thereby supporting productivity, sustainability, and profitability in the global swine industry.

## 2. Materials and Methods

### 2.1. Animals and Phenotype Data

A total of 51,028, 14,378, and 7255 records corresponding to ten reproductive traits were collected from 15,082 Yorkshire, 4828 Landrace, and 2287 Duroc pigs, respectively. These animals were raised under uniform feeding and management conditions across two farms located in Chifeng, Inner Mongolia, China. Pigs in both farm units were managed under an identical feeding regimen with a consistent feed formulation and maintained a uniformly high health status. Both farms have undergone rigorous and continuous health monitoring and have been confirmed as negative for multiple respiratory diseases and diarrhea pathogens. This standardized management effectively eliminates potential confounding effects of nutritional variation and health status on reproductive performance, thereby enhancing the reliability and comparability of subsequent genetic analyses. The ten traits included TNB, NBA, number born healthy (NBH), number of mummies (NM), number born weak (NBW), number of stillbirth (NS), gestation length (GL), litter weight (LW), survival rate (SR), and uniformity (CV). The number of piglets born healthy and weak indicated those with birth weights of >0.8 kg and <0.8 kg, respectively. These records spanned 1–11 parities.

The SR and CV were calculated using the following formulas:

$$SR=\frac{NBA}{TNB}\times 100\%,$$

and

$$uniformity(CV)=\frac{Standard\;deviation\;of\;birth\;weight\;of\;piglets\;in\;the\;same\;litter}{Average\;birth\;weight\;of\;piglets\;in\;the\;same\;litter}.$$

### 2.2. Estimation of Genetic Parameters

Genetic parameters and breeding values for reproductive traits were estimated using the ASReml V4.1.0 software, employing a repeatability animal model expressed as follows:

y=Xb+Za+Wpe+e.

In this model, y represents the vector of the phenotype for each reproductive trait; b is the vector of fixed effects, including farm (1–2), batch (1–384), and parity (1–11); a is the vector of additive genetic effect; and pe is the vector of permanent environmental effect. In our repeatability animal model, the permanent environmental effect (pe) was included to account for non-genetic influences that consistently affect repeated reproductive records of the same sow across different parities. Because each sow had multiple records collected over time, the permanent environmental effect captures individual-specific environmental influences that persist across parities. X, Z, and W are the corresponding incidence matrices, and e represents the residual effect.

The following genetic parameters were calculated:

$$h^{2}=\frac{\sigma_{a}^{2}}{\sigma_{a}^{2}+\sigma_{pe}^{2}+\sigma_{e}^{2}}\;h_{pe}^{2}=\frac{\sigma_{a}^{2}+\sigma_{pe}^{2}}{\sigma_{a}^{2}+\sigma_{pe}^{2}+\sigma_{e}^{2}}$$

where $h^{2}$ denotes heritability; $h_{pe}^{2}$ represents repeatability; $\sigma_{a}^{2}$ is the additive genetic variance; $\sigma_{pe}^{2}$ indicates permanent environmental effect variance; and $\sigma_{e}^{2}$ is the residual variance.

### 2.3. Genotyping and Quality Control

Genotyping for GWAS and genomic prediction was conducted using three different chips: KPS^®^ Zhongxin-1 Porcine Breeding ChipV2 (51,315 SNPs, Beijing Compass, Beijing, China), KPS^®^ Zhongxin-1 Porcine Breeding Chip_plus (57,466 SNPs, Beijing Compass, Beijing, China), and CAU50K [21]. Details regarding sample collection, chip measurement, merging procedures, and quality control have been previously reported in our study [22]. Genotype data from the three chips were merged and quality-controlled using PLINK (https://www.cog-genomics.org/plink/, accessed on 16 March 2026). Sex-chromosome loci were excluded, and only autosomal SNPs were retained for further analyses. Individuals with a call rate < 80%, SNPs with a missing rate > 10%, and SNPs with a minor allele frequency ≤ 0.05 were removed. After quality control, a total of 46,358 SNPs were used for subsequent analyses in three breeds including 6235 Yorkshire, 1807 Landrace, and 1129 Duroc pigs.

### 2.4. Genome-Wide Association Study (GWAS)

The GWAS for the ten reproductive traits was conducted using GCTA 1.94.1 software within the following model:

y= μ+bX+G+e

where y is the vector of estimated breeding values for all individuals across the aforementioned ten traits; b represents the average effects of gene substitution of for a specific SNP and X denotes the vector of the SNP genotype; G indicates the genomic relationship matrix created by all SNPs; and e denotes the random residual.

A Bonferroni correction threshold of 0.05/46,358 (*p*-value < 1.0786 × 10^−6^) was applied, and SNPs with a *p*-value below this threshold were considered significant. GWAS results were visualized using Manhattan and quantile–quantile plots, in which the *Y*-axis represented the −log10-transformed *p*-values for SNPs.

Additionally, candidate genes within 1 million base pairs (1 Mb) of significant SNPs were identified using BioMart 0.10.1. The pig reference genome (Sscrofa11.1) was used for this analysis. Functional annotation of the candidate genes was performed using the KOBAS tool (http://bioinfo.org/kobas/annotate/, accessed on 5 July 2024) [23], and gene enrichment with a *p*-value < 0.05 was considered statistically significant.

### 2.5. Genomic Prediction

The GBLUP method utilizes genetic relationships to assess the genetic potential of individuals [24]. In this study, genomic prediction was performed using the GBLUP with ASReml V4.1.0 software. The model was specified as follows:

y=1μ+Zg+e

where y is the vector of phenotypic values, g is the vector of additive genetic values, Z represents the incidence matrix of g, and e is the vector of residual effect. Genomic marker information derived from SNPs was used to compute the GBLUP.

We evaluated the accuracy of genomic prediction using five-fold cross-validation. The dataset was randomly divided into five equal-sized subsets. In each fold, one subset was used as the validation set, and the other four served as the reference population for model training. Prediction accuracy was defined as the Pearson correlation between adjusted phenotypic values and genomic estimated breeding values in the validation set. Each subset was validated exactly once, and the final reliability was represented by the average correlation across all five folds.

## 3. Results

### 3.1. Phenotypic Description and Genetic Parameter Estimation

Descriptive statistics for the ten key reproductive traits were calculated, and the results are presented in Table 1. The ten traits exhibited substantial variation across the three breeds. For reproductive traits, Yorkshire generally outperformed Landrace and Duroc, with Duroc exhibiting the lowest reproductive performance among the three breeds. For example, the TBA (total born alive) in Yorkshire was 15.27 ± 3.66, in Landrace it was 13.33 ± 3.12, and in Duroc it was 10.18 ± 2.74. Genetic parameters were estimated using both phenotypic and pedigree data, with the findings summarized in Table 2. The heritability estimates for the ten traits were moderately low across the three breeds. Specifically, heritability values ranged as follows: TNB: 0.09–0.13, NBA: 0.09–0.11, NBH: 0.07–0.15, NM: 0.01–0.02, NBW: 0–0.24, NS: 0.01–0.08, GL: 0.33–0.41, LW: 0.10–0.15, SR: 0.02–0.07, and CV: 0.01–0.11. Among these, GL exhibited the highest heritability, while NM showed the lowest. Additionally, the repeatabilities of ten reproductive traits were estimated, with values ranging from 0 to 0.09. Such low repeatability indicates that performance in one parity provides limited information about future reproductive performance, highlighting the importance of multiple records per sow to improve the accuracy of genetic evaluation. 

### 3.2. Genome-Wide Association Study (GWAS) of the Ten Key Reproductive Traits

**Figure 1:**
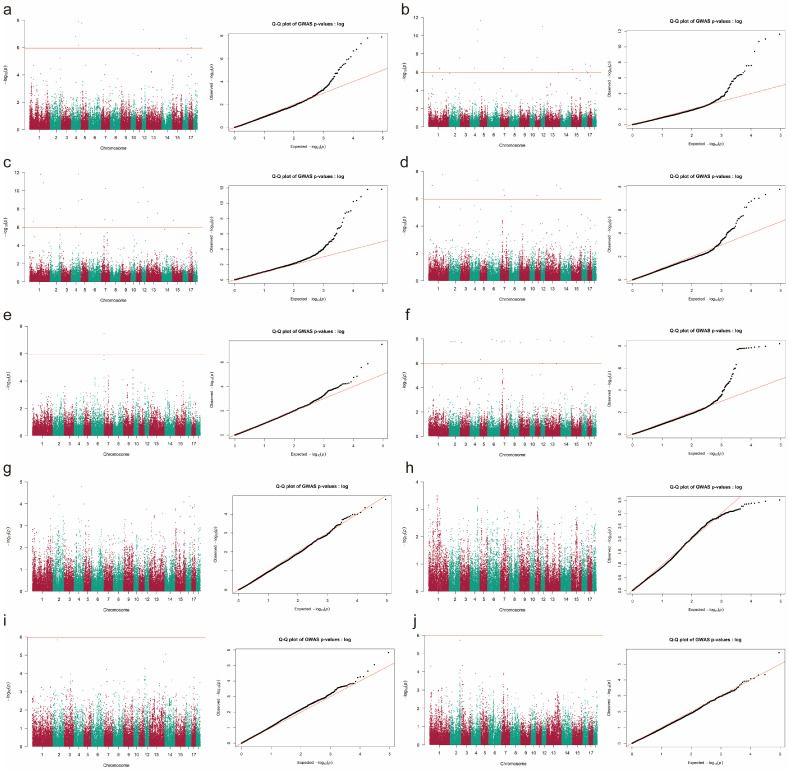
GWAS results of reproductive traits in Yorkshire pigs. The *Y*-axis represents the −log10-transformed *p*-values for SNPs. −log10(*P*) = Observed −log10(*P*). The results of (**a**–**j**) indicate the Manhattan and Q-Q plots of GWAS for TNB, NBH, NBW, NS, GL, SR, NBA, NM, LW and CV, respectively. TNB = total number born, NBA = number born alive, NBH = number born healthy, NM = number of mummies, NBW = number born weak, NS = number of stillbirth, GL = gestation length, LW = litter weight, SR = survival rate, CV = uniformity.

A GWAS for reproductive traits was conducted using GCTA software. A total of 37 genome-wide significant SNPs, located on 17 different Sus scrofa chromosomes (SSCs), were identified for seven reproductive traits in Yorkshire pigs (Figure 1 and Table S1). Specifically, the following results were observed. TNB: seven significant SNPs were identified on SSC4, SSC5, SSC12, SSC16, and SSC17 (*P* < 1.06 × 10^−6^); NBH: 15 significant SNPs were identified on SSC1, SSC2, SSC4, SSC5, SSC7, SSC10, SSC11, SSC12, SSC15, SSC16, and SSC17 (*P* < 8.05 × 10^−7^); NBW: 17 significant SNPs were identified on SSC1, SSC2, SSC4, SSC5, SSC7, SSC8, SSC11, SSC12, SSC13, and SSC15 (*P* < 8.40 × 10^−7^); NS: eight significant SNPs were identified on SSC1, SSC4, SSC7, SSC11, and SSC13 (*P* < 5.95 × 10^−7^); SR: 17 significant SNPs were identified on. SSC2, SSC3, SSC5, SSC6, SSC7, SSC8, SSC9, SSC11, SSC12, SSC13, SSC14, and SSC17 (*P* < 1.04 × 10^−6^); GL: one significant SNP was detected on SSC6 (*P* = 3.54 × 10^−8^). Among these, SNP CNC10042060 was significantly associated with TNB, NBH, NBW and NS, and SNP CNC10041283 strongly impacted TNB, NBH and NBW. Additionally, SNPs CNC10120451 and CNCB10003799 were significantly associated with TNB, NBH, NBW and SR, and CNCB10007759 was strongly associated with NBH, NBW, NS and SR.

**Figure 2:**
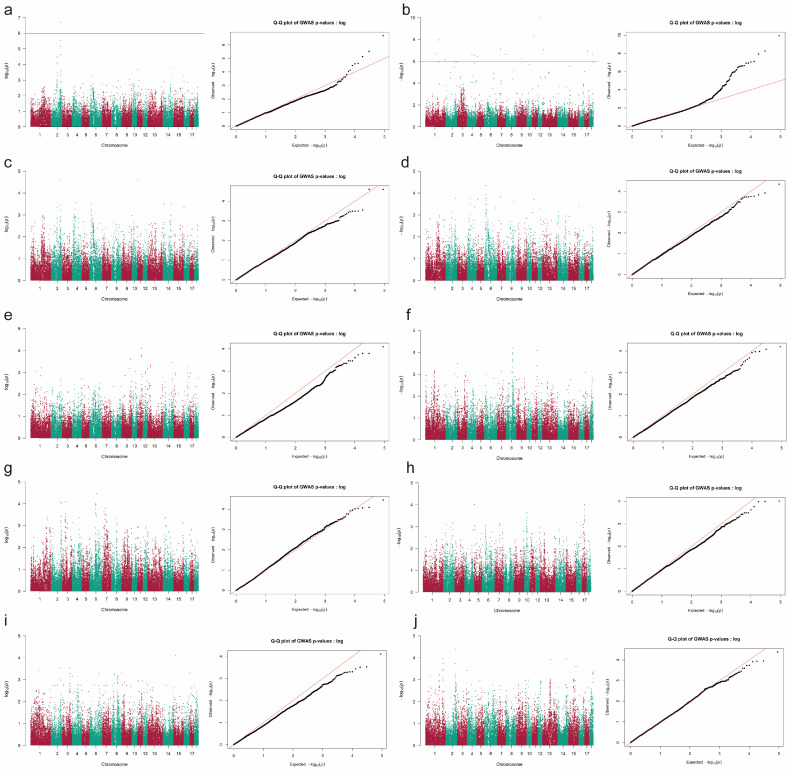
GWAS result of reproductive traits in Landrace pigs. The *Y*-axis represents the −log10-transformed *p*-values for SNPs. −log10(*P*) = Observed −log10(*P*). The results of (**a**–**j**) indicate the Manhattan and Q-Q plots of GWAS for TNB, NBW, NBA, NBH, NM, NS, GL, LW, SR, and CV, respectively. TNB = total number born, NBA = number born alive, NBH = number born healthy, NM = number of mummies, NBW = number born weak, NS = number of stillbirth, GL = gestation length, LW = litter weight, SR = survival rate, CV = uniformity.

**Figure 3:**
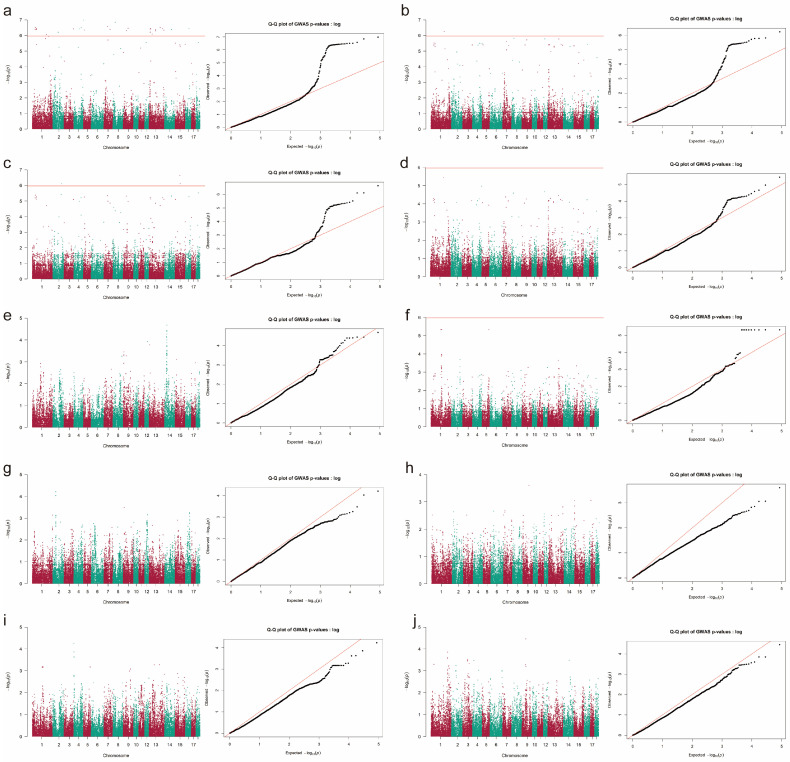
GWAS result of reproductive traits in Duroc pigs. The *Y*-axis represents the −log10-transformed *p*-values for SNPs. −log10(*P*)=Observed −log10(*P*). The results of (**a**–**j**) indicate the Manhattan and Q-Q plots of GWAS for TNB, NBH, NBW, NBA, NM, NS, GL, LW, SR, and CV, respectively. TNB = total number born, NBA = number born alive, NBH = number born healthy, NM = number of mummies, NBW = number born weak, NS = number of stillbirth, GL = gestation length, LW = litter weight, SR = survival rate, CV = uniformity. The orange horizontal line indicated a *p*-value < 1.0786 × 10^−6^.

For Landrace pigs, 16 SNPs (Figure 2 and Table S1) located on 11 SSCs were significantly associated with TNB (*P* = 1.01 × 10^−6^~1.12 × 10^−10^), and one SNP on SSC2 was strongly associated with NBW (*P* = 2.08 × 10^−7^). In Duroc pigs, a total of 31 significant SNPs (Figure 3 and Table S1) were detected across 12 SSCs (*P* = 1.02 × 10^−7^~8.58 × 10^−7^), with 27 SNPs strongly associated with TNB, one SNP with NBH, and three SNPs with NBW. Three SNPs, CNC10042060, CNC10160995, and CNCB10003799, were significantly associated with TNB both in Yorkshire and Duroc pigs. Five SNPs, CNC10012965, CNC10042060, CNC10120451, CNCB10003799, and CNCB10007759, showed strong associations with NBW in Yorkshire and Landrace pigs.

### 3.3. Candidate Gene Identification and Functional Annotation

A total of 1180 candidate genes were identified by screening the ±1 Mb flanking regions of 37 significant SNPs of Yorkshire pigs (Table S2). Functional annotation of these genes was performed using the KOBAS tool (http://bioinfo.org/kobas/annotate/, accessed on 5 July 2024) [23], revealing that 211 genes were involved in 15 significant KEGG pathways and 62 strong GO terms (*P* < 0.05, Table S3). The most significant KEGG pathway was the estrogen signaling pathway (ssc04915, *P* = 1.19 × 10^−10^). Notable GO terms included cell division and metabolism processes, such as the regulation of cytokine production (GO:0001817), regulation of cell division (GO:0051302), and glutathione metabolic process (GO:0006749).

For Landrace pigs, 523 candidate genes were detected (Table S2), with functional annotation showing that 126 genes were involved in 12 KEGG pathways and 85 strong GO terms (*P* < 0.05, Table S4). The estrogen signaling pathway (ssc04915, *P* = 5.30 × 10^−16^) was again the most significant (ssc04915, *P* = 5.30 × 10^−16^). The GO terms mainly were related to embryonic development, for example, embryonic skeletal system morphogenesis (GO:0048704), embryonic forelimb morphogenesis (GO:0035115), and embryonic limb morphogenesis (GO:0030326).

For Duroc pigs, 680 candidate genes were identified (Table S2), with 110 genes involved in 71 significant enrichments (*P* < 0.05, Table S5), including four KEGG pathways and 66 GO terms. The identified KEGG pathways were acute myeloid leukemia (ssc05221), the PPAR signaling pathway (ssc03320), nucleotide excision repair (ssc03420), and the neurotrophin signaling pathway (ssc04722). Go terms were mainly related to embryonic development, DNA replication, and cell proliferation, such as in utero embryonic development (GO:0001701), embryonic pattern specification (GO:0009880), DNA replication-dependent nucleosome assembly (GO:0006335), DNA clamp loader activity (GO:0003689), negative regulation of cyclin-dependent protein serine/threonine kinase activity (GO:0045736), and positive regulation of stem cell proliferation (GO:2000648).

### 3.4. Genomic Prediction for Key Reproductive Traits

In Yorkshire pigs, genomic prediction accuracy for reproductive traits ranged from 0.21 to 0.68 (Table 3). The genomic prediction accuracy was relatively high for the GL (0.68 ± 0.02), NBW (0.54 ± 0.04), and LW (0.52 ± 0.02). The NS, SR, NBH, and TNB had moderate genomic prediction accuracies, namely, 0.46 ± 0.01, 0.43 ± 0.02, 0.42 ± 0.01 and 0.40 ± 0.01. The genomic prediction accuracies of NBA and CV were 0.34 ± 0.01 and 0.30 ± 0.02, respectively. The NM had the lowest genomic prediction accuracy with 0.21 ± 0.02.

For Landrace pigs, the genomic prediction accuracy for reproductive traits ranged from 0.25 to 0.59 (Table 3). The GL and NBW had relatively high genomic prediction accuracies with 0.59 ± 0.03 and 0.54 ± 0.03, respectively. Moderate genomic prediction accuracies were found for TNB (0.43 ± 0.04), NBH (0.43 ± 0.04), NS (0.42 ± 0.03) and NBA (0.41 ± 0.03). The genomic prediction accuracies for LW and NM were 0.33 ± 0.05 and 0.25 ± 0.01, respectively. SR and CV had the same genomic prediction accuracy of 0.39.

In Duroc pigs, genomic prediction accuracy for reproductive traits ranged from 0 to 0.57 (Table 3). The highest genomic prediction accuracy was observed for GL (0.57 ± 0.06). The TNB, NBA, and NBH had moderate genomic prediction accuracies, namely, 0.48 ± 0.09, 0.42 ± 0.07, and 0.44 ± 0.06. The genomic prediction accuracies of NBW, NS, SR, LW and NM were 0.39 ± 0.04, 0.34 ± 0.05, 0.33 ± 0.07, 0.29 ± 0.03 and 0.22 ± 0.01, respectively. CV had the lowest genomic prediction accuracy at 0. This finding can be attributed to several non-mutually exclusive factors. First, as a low-heritability trait, the accuracy of genomic prediction is highly sensitive to reference population size; the limited number of Duroc records may have provided insufficient statistical power to distinguish true genetic signals from residual noise using GBLUP. Second, it is plausible that genetic variation for this specific trait has been largely exhausted in the Duroc population due to prior directional selection or a founder effect, resulting in negligible additive genetic variance. Third, CV traits are complex and may be governed by non-additive genetic effects (e.g., dominance or epistasis) that are not captured by the additive GBLUP model. This result underscores the need for larger Duroc-specific reference populations or alternative models to dissect the genetic architecture of CV traits in this breed.

## 4. Discussion

This study aimed to estimate the genetic parameters for key reproductive traits in three major commercial pig breeds, Yorkshire, Landrace, and Duroc, and identify genomic loci associated with these traits. In addition, we also evaluate the genomic prediction accuracy for these traits. Yorkshire, Landrace, and Duroc constitute the primary breeds driving global pig production. Our results illuminate the genetic architecture of porcine reproduction, revealing both breed-specific and shared determinants of reproductive performance that can inform breeding strategies to enhance productivity and profitability.

The three breeds have comparatively low to moderate heritability estimates for reproductive features, which is in line with earlier research on pig reproductive traits [25,26]. In particular, the heritability values for NBA, TNB, and other important characteristics including LW and GL varied from 0 to 0.41. These findings demonstrate the intricacy of genetic regulation of reproductive performance, as well as the impact of environmental conditions, which are known to affect pig reproductive success. While there might have been some consistency in reproductive success across parities, the repeatability estimates, which ranged from 0 to 0.09, were similarly low for the majority of traits, indicating that the attributes were subject to significant environmental variation. The significance of taking both genetic and environmental factors into account when making breeding decisions was further highlighted by these repeatability estimates. In this study, the heritability estimates were generally consistent with those reported in previous studies. However, differences among breeds were observed. Such variation may be attributed to differences in genetic background, which can influence the magnitude of additive genetic variance for the traits studied. In addition, environmental and management factors, including herd management practices, nutrition, and parity structure, may contribute to differences in phenotypic variance across breeds. Differences in sample size and population structure may also affect the accuracy of variance component estimation.

A number of important SNPs linked to reproductive characteristics in all three breeds were found by the genome-wide association study (GWAS). These findings reinforced the potential of these genomic regions as key candidates for improving reproductive efficiency in commercial pig breeding. We identified 84 candidate genes shared by all three breeds. These overlapping genes represent high-priority targets for genetic improvement, as they may carry causal variants with consistent effects across populations. Of these, 53 could be annotated with gene names. Some genes had been reported to be associated with reproductive traits and development in pigs, such as TXNIP [27], FMO5 [28], CHD1L [29], SEC22B [30], CKAP4 [31], and SYN3, FBXO7, BPIFC, and RTCB [32]. The functional annotation of these candidate genes revealed several biologically relevant pathways involved in reproduction, such as the estrogen signaling pathway. Estrogen signaling is a crucial regulator of reproductive function, influencing establishment of pregnancy [33,34] and uterine gland development [35].

Genomic prediction accuracy for reproductive traits varied across the three breeds, with moderate to high accuracies observed for some traits in all breeds. For example, in Yorkshire pigs, the accuracy for GL was relatively high (0.68), suggesting that this trait was more genetically predictable and could be more easily selected for in breeding programs. Similarly, traits such as NBW and LW also showed moderate prediction accuracy, reflecting the potential to improve these traits through genomic selection. However, several traits, such as NM, showed lower genomic prediction accuracy, indicating that these traits were either influenced by a larger environmental component or had a more complex genetic architecture. In addition, model specification may limit the detection of genetic effects. Phenotype quality and consistency also influence accuracy, especially for hard-to-measure traits strongly affected by management and environment. This suggests that genomic prediction models may need to be refined, possibly by incorporating more detailed environmental data or by using multi-breed approaches to improve prediction accuracy. In Landrace and Duroc pigs, genomic prediction accuracy of these traits could also be used to enhance this understanding. The identification of key candidate genes and genomic regions associated with reproductive traits offers actionable insights for improving genetic gain through genomic selection. However, the relatively low heritability and genomic prediction accuracies for some traits suggest that additional research is needed to refine genomic prediction models and to explore the potential for crossbreeding and multi-breed genomic approaches to enhance reproductive performance. Furthermore, incorporating more precise environmental data and investigating genotype-by-environment interactions could improve the accuracy of genomic prediction models, especially for traits with low heritability and genomic prediction accuracy.

These observed accuracies could inform breed-specific selection indices. Adding multi-level information and using different methods can improve the accuracy of genomic prediction of traits [19,36,37,38,39,40,41]. In future studies, prediction models should be refined, e.g., by incorporating richer environmental covariates, explicitly modeling genotype-by-environment interactions, and leveraging multi-breed training sets to improve cross-population performance. Moreover, the identified SNPs and candidate genes provide actionable targets for genomic selection to increase genetic gain.

## 5. Conclusions

This study investigated the genetic architecture of ten reproductive traits in three major commercial pig breeds (Yorkshire, Landrace, and Duroc) using GWAS and genomic prediction. Heritability estimates ranged from low to moderate, with gestation length showing the highest values and number of mummies the lowest. Several genome-wide significant loci and candidate genes associated with reproductive traits were identified, including shared SNPs associated with total number born and newborn weight across breeds. Genomic prediction accuracies varied among traits and populations, reflecting the complex genetic architecture of reproductive traits. However, the relatively small sample sizes for some breeds and the low heritability of certain traits might have limited the detection of loci with small effects. Overall, these findings provide insights into the genetic basis of reproductive performance and offer potential markers for genomic selection in pig breeding programs. In future studies, functional validation of key candidate genes in independent or progeny populations is needed to facilitate their application in breeding programs.

## Figures and Tables

**Table 1:** Descriptive statistics of ten key reproduction traits in Yorkshire, Landrace, and Duroc pigs.

	Yorkshire Pigs			Landrace Pigs			Duroc Pigs		
Traits	Mean	SD	Records	Mean	SD	Records	Mean	SD	Records
TNB	15.27	3.66	50,764	13.33	3.12	14,295	10.18	2.74	7235
NBA	13.72	3.44	50,403	12.17	2.95	14,217	9.36	2.62	7122
NBH	13.35	3.41	50,318	11.93	2.95	14,196	9.22	2.63	7118
NM	0.26	0.55	49,775	0.23	0.52	14,122	0.18	0.46	7179
NBW	0.38	0.87	49,994	0.18	0.48	13,919	0.07	0.26	7008
NS	1.03	1.29	50,014	0.81	1.09	14,181	0.61	0.90	7103
GL	115.63	1.38	50,594	116.62	1.31	14,198	115.72	1.24	7175
LW	13.58	9.04	51,026	14.16	7.99	14,376	12.19	6.97	7255
SR	90.58	10.28	50,126	91.93	9.35	14,137	91.95	10.83	7089
CV	0.22	0.08	39,310	0.21	0.08	12,114	0.18	0.09	6161

Note: TNB = total number born, NBA = number born alive, NBH = number born healthy, NM = number of mummies, NBW = number born weak, NS = number of stillbirth, GL = gestation length, LW = litter weight, SR = survival rate, CV = uniformity, and SD = standard deviation. The number of piglets born healthy and weak indicates those with birth weights of >0.8 kg and <0.8 kg, respectively.

**Table 2:** Genetic parameters of ten key reproduction traits in Yorkshire, Landrace, and Duroc pigs.

	Yorkshire Pigs				Landrace Pigs				Duroc Pigs			
Traits	*h* ^2^	SE of *h*^2^	$h_{pe}^{2}$	SE of $h_{pe}^{2}$	*h* ^2^	SE of *h*^2^	$h_{pe}^{2}$	SE of $h_{pe}^{2}$	*h* ^2^	SE of *h*^2^	$h_{pe}^{2}$	SE of $h_{pe}^{2}$
TNB	0.13	0.01	0.09	0.01	0.09	0.01	0.08	0.01	0.11	0.02	0.08	0.02
NBA	0.11	0.01	0.09	0.01	0.09	0.01	0.08	0.01	0.10	0.02	0.08	0.02
NBH	0.15	0.01	0.07	0.01	0.07	0.01	0.07	0.01	0.14	0.02	0.07	0.02
NM	0.02	0.00	0.01	0.00	0.01	0.00	0.01	0.01	0.01	0.01	0.00	0.01
NBW	0.24	0.01	0.00	0.00	0.00	0.00	0.00	0.00	0.15	0.01	0.00	0.00
NS	0.08	0.01	0.01	0.01	0.01	0.01	0.01	0.01	0.07	0.01	0.00	0.01
GL	0.41	0.01	0.04	0.01	0.33	0.02	0.01	0.01	0.37	0.03	0.03	0.02
LW	0.15	0.00	0.00	0.00	0.10	0.01	0.00	0.00	0.15	0.02	0.00	0.02
SR	0.07	0.01	0.02	0.01	0.02	0.01	0.02	0.01	0.07	0.01	0.00	0.00
CV	0.11	0.01	0.01	0.01	0.01	0.01	0.03	0.01	0.02	0.01	0.02	0.01

Note: TNB = total number born, NBA = number born alive, NBH = number born healthy, NM = number of mummies, NBW = number born weak, NS = number of stillbirth, GL = gestation length, LW = litter weight, SR = survival rate, CV = uniformity, and SE = standard error. The number of piglets born healthy and weak indicates those with birth weights of >0.8 kg and <0.8 kg, respectively.

**Table 3:** GBLUP method for genomic prediction accuracy analysis of reproductive traits in Yorkshire, Landrace, and Duroc pigs.

Traits	Accuracy of Genomic Prediction in Yorkshire Pigs	SD	Accuracy of Genomic Prediction in Landrace Pigs	SD	Accuracy of Genomic Prediction in Duroc Pigs	SD
TNB	0.40	0.01	0.43	0.04	0.48	0.09
NBA	0.34	0.01	0.41	0.03	0.42	0.07
NBH	0.42	0.01	0.43	0.04	0.44	0.06
NM	0.21	0.02	0.25	0.01	0.22	0.01
LW	0.52	0.02	0.33	0.05	0.29	0.03
NBW	0.54	0.04	0.54	0.03	0.39	0.04
NS	0.46	0.01	0.42	0.03	0.34	0.05
SR	0.43	0.02	0.39	0.04	0.33	0.07
CV	0.30	0.02	0.39	0.02	0.00	0.00
GL	0.68	0.02	0.59	0.03	0.57	0.06

Note: TNB = total number born, NBA = number born alive, NBH = number born healthy, NM = number of mummies, NBW = number born weak, NS = number of stillbirth, GL = gestation length, LW = litter weight, SR = survival rate, CV = uniformity, and SD = standard deviation.

## Data Availability

Data will be made available in this manuscript. The genotype and phenotype used for breeding could be obtained from the corresponding author via reasonable request.

## Supplementary Materials

The following supporting information can be downloaded at https://www.mdpi.com/article/10.3390/ani16070999/s1, Table S1: Significant SNPs associated with reproductive traits in Yorkshire, Landrace, and Duroc pigs; Table S2: Candidate genes by screening the ±1 Mb flanking regions of significant SNPs; Table S3: Functional annotation of candidate genes of reproductive traits in Yorkshire pigs; Table S4: Functional annotation of candidate genes of reproductive traits in Landrace pigs; Table S5: Functional annotation of candidate genes of reproductive traits in Duroc pigs.
